# Refractory hyponatremia in neuromyelitis optica in a pediatric patient: A case report

**DOI:** 10.1097/MD.0000000000026231

**Published:** 2021-07-23

**Authors:** Tai-Han Lin, Po-Chang Hsu, Chia-Cheng Sung, Hung-Hsiang Fang, Chiung-Hsi Tien, Chih-Fen Hu, Po-Wei Wu, Chia-Hsiang Yu, Jhao-Jhuang Ding, Sheng-Yuan Ho, Shyi-Jou Chen

**Affiliations:** a Department of Pediatrics, Tri-Service General Hospital, National Defense Medical Center, No.325, Section 2, Chenggong Rd., Neihu District, Taipei, Taiwan; b Graduate Institute of Medical Sciences, National Defense Medical Center, No. 161, Section 6,MinChuan East Road, Neihu, Taipei, Taiwan; c Department of Microbiology and Immunology, National Defense Medical Center, No. 161, Section 6, MinChuan East Road, Neihu, Taipei, Taiwan.

**Keywords:** case report, NMO, refractory hyponatremia, SIADH

## Abstract

**Rationale:**

Neuromyelitis optica spectrum disorders (NMOSD) is a rare autoimmune disease predominantly involving optic nerves and spinal cord, and possible comorbidities including syndrome of inappropriate antidiuretic hormone secretion or urinary complication. We reported a young girl diagnosed with NMOSD presented with refractory hyponatremia, acute urine retention, and general weakness. Clinical symptoms improved gradually after receiving intravenous immunoglobulin, high-dose methylprednisolone, and plasmapheresis. NMOSD should be kept in mind in adolescence with acute urine retention, intermittent fever, and hyponatremia.

**Patient concerns:**

A 15-year-old girl admitted to our hospital due to no urination for 2 days.

**Diagnosis:**

Aquaporin-4 antibodies were detected showing positive both in serum and cerebrospinal fluid. Long transverse myelitis in cervical and thoracic spinal cord and optic neuritis was revealed in magnetic resonance imaging.

**Interventions:**

Intravenous immunoglobulin 2 g/kg was infused totally in 4 days, and methylprednisolone pulse therapy was subsequently followed in 5 days; followed by 5 courses of plasmapheresis a week later.

**Outcomes:**

Her muscle power, syndrome of inappropriate antidiuretic hormone secretion condition, and urinary function were all improved after immune-modulated treatment course; NMOSD relapsed twice within the first year after diagnosis, however no relapse of NMOSD in the subsequent 1 year.

**Lessons:**

To the best of our knowledge, this was the first childhood case of NMO accompanied by refractory hyponatremia in the reported literature. In childhood cases presenting with refractory hyponatremia and limb weakness, NMO or NMOSD should be considered possible diagnoses despite their rarity in pediatric cases.

## 1 Introduction

Neuromyelitis optica (NMO) is a rare inflammatory and autoimmune demyelinating disease predominantly involving the optic nerves and spinal cord. Spinal injury caused by myelitis could be a reasonable explanation for urinary complication.^[1]^ Moreover, syndrome of inappropriate antidiuretic hormone secretion (SIADH) or other autoimmune diseases have been reported as comorbidities of NMO.^[1,2]^ However, almost all cases of NMO or neuromyelitis optica spectrum disorder (NMOSD) associated with SIADH and hyponatremia and urinary complication are reported in adulthood. Herein, we report an unusual childhood case diagnosed as typical NMO accompanied by the rare presentation of refractory hyponatremia as initial symptoms before diagnosis of SIADH caused by NMO.

## 2 Case report

A 15-year-old girl admitted to our hospital due to no urination for 2 days. She had fever intermittently 1 week prior to admission. Although the fever subsided after 1 day, she gradually developed nausea, poor appetite, abdominal pain, generalized weakness, and bilateral lower limb numbness over the 6-day period prior to presenting at the hospital.

**Figure 1:**
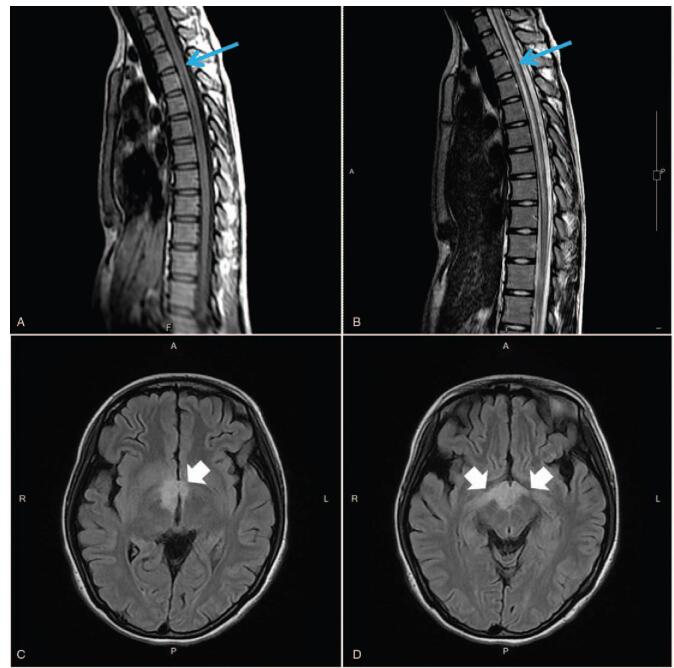
Spinal MRI findings (arrows) indicating transverse myelitis. A, T1 sagittal. B, T2 sagittal with high signal through spinal canal at C6-T9 level. Brain MRI findings (arrows). C, T2 axial with hyperintensity in hypothalamic region. D, T2 axial with hyperintensity in hypothalamic region and bilateral post-chiastic optic tract.

The admission procedure revealed reduced muscle power and deep tendon reflex in the bilateral lower extremities without pitting edema; neither tachycardia nor dry mucous was presented. A urinary catheter was inserted due to urine retention. Unpredictably, she exhibited severe hyponatremia with serum sodium (107 mmol/L), together with significant low plasma osmotic pressure (222 mOsm/kg), concentrated urine (798 mOsm/kg), and sodium in the urine (87 mmol/L). Examinations were performed to determine the cause of possible SIADH.^[2]^ In addition, a fluid restriction strategy (within 1 L) was imposed while cautiously correcting the sodium level. Regarding the endocrine function, the patient presented with normal secondary sex characteristics development and a normal thyroid function. However, she exhibited an elevated level of somatotropin and prolactin, together with the hyposecretion of estradiol and luteinizing hormone. Central original lesions in the hypothalamic area were thus considered, and brain magnetic resonance imaging (MRI) was arranged subsequently. Brain and spinal cord MRI T2-weighted images revealed multiple hyperintense lesions in the cervical and thoracic spinal cord (Fig. 1A, B), hypothalamic region (Fig. 1C), and bilateral post-chiastic optic tract with edematous change (Fig. 1D).

The patient showed a facial malar rash and the progressive development of lethargy and weakness. Moreover, the Babinski reflex was found in the bilateral lower limbs. Bilateral retrobulbar neuritis was further diagnosed by an ophthalmologist, together with impaired color differentiation and vision. Thus, aquaporin-4 antibodies (AQP4-Ab) were detected in both the patient's serum and cerebrospinal fluid via a cell-based method (Anti-Aquaporin-4 IIFT EUROIMMUN Co) Accordingly, a diagnosis of NMO was confirmed.^[3]^

**Figure 2:**
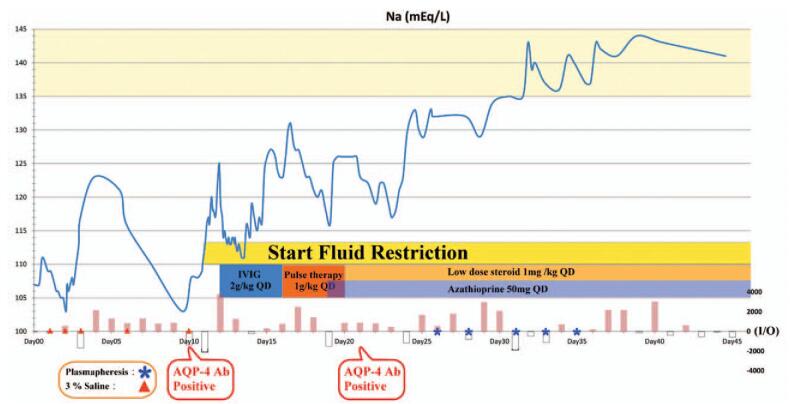
Clinical course. IVIG 2 g/kg was infused during days 12–15, and methylprednisolone pulse therapy was subsequently performed during days 16–20. AQP4-Ab rechecked on day 21 still remained positive. Consequently, 5 courses of plasmapheresis were arranged since on days 26. The patient's muscle power and SIADH condition improved gradually following treatment and follow-up management. Input and output statuses were depicted in the lowest part. AQP4-Ab = aquaporin-4 antibodies, IVIG = intravenous immunoglobulin, SIADH = syndrome of inappropriate antidiuretic hormone secretion.

She was treated with intravenous immunoglobulin (IVIG) (500 mg/kg/d for 4 days) followed by steroid pulse therapy with high-dose methylprednisolone (1000 mg/kg/d) for a further 5 days. However, she still exhibited persistent refractory hyponatremia, a positive aquaporin-4 antibodies (AQP4-Ab) result in rechecked serum, and only partial neurological improvement. Presentation of limbs weakness and hyponatremia improved gradually after she was treated with 5 courses of plasmapheresis. Afterward, she was administrated with oral prednisolone (0.5–1 mg/kg/d, every other day) and azathioprine 100 mg (2 mg/kg/d). The sodium variation was gradually brought under control, as shown in Figure 2, and was accompanied by an improvement in the muscle power, fever, SIADH condition, and urinary function. Her gradual improvement was reflected in an EDSS recovery from 9 to 3. However, the disease relapsed twice at 5 months and 7 months with presentation of limbs numbness and intermittent convulsion after first time of remission. The patient did not suffer from disease relapsed in the subsequent 1 year.

## 3 Discussion

NMOSD is a rare autoimmune disorder involving central nervous system predominantly affecting female patients.^[4]^ The documented prevalence rate of NMOSD ranged between 5–100 per 1,000,000 population.^[4]^ The majority cases of NMOSD were found positive for AQP4-Ab; while others were found positive for myelin oligodendrocyte glycoprotein antibodies (MOG-Ab).^[5]^ The recorded female-to-male ratio was up to 9 with mean onset at age 40 for patients with AQP4-Ab, while the sex ratio was around 1 for patients with MOG-Ab.^[4]^ Mortality rates of NMOSD patients ranged between 9% and 32% worldwide, and patients with MOG-Ab showed favorable prognosis than those with AQP4-Ab.^[5,6]^

The diagnostic criteria of NMOSD with AQP4-Ab including at least one major clinical characteristic (optic neuritis, acute myelitis, area postrema syndrome, brainstem syndrome, or symptomatic syndrome with typical MRI lesions), detectable AQP4-Ab, and exclusion of possible similar diagnosis (malignancy, sarcoidosis, chronic infection, and so forth).^[7,8]^ Featured MRI lesions could involve diencephalic, cerebral, dorsal brainstem, periventricular, dorsal medulla, or ≥ 3 consecutive spinal cords.^[8,9]^ Multiple sclerosis demonstrate better outcome compared to NMOSD despite they could be confused sometimes; they can be differentiated by lumbar puncture, presence of coexisted autoimmune disease, disease severity, or involved patterns under MRI imaging.^[8]^ Treatment for NMO should be initiated to avoid further disability, and the goal is to minimize disease relapsing.^[10]^ Therapeutic options included pulse therapy, plasmapheresis, IVIG, rituximab, or other immunosuppressive agents.^[10,11]^ Clinical and MRI findings were compatible with the diagnostic criteria of NMOSD with AQP4-Ab in our patient. Our patient showed presence of AQP4-Ab and absence of MOG-Ab. She suffered from symptoms of optic neuritis and acute myelitis with corresponding involvement of brain and spinal lesion under MRI imaging. Our patient was treated with IVIG, pulse therapy, and plasmapheresis during acute phase; oral prednisolone and azathioprine as maintenance therapy.

Our patient presented with acute urine retention (AUR), together with general weakness and intractable hyponatremia, and was impressed as SIADH initially. NMO was diagnosed after serial examination. AUR is less common in women compared to men, and possible cause including neurological, anatomical, myopathic, infective, pharmacological, or functional.^[12]^ It was reported that urinary complication was more severe in NMOSD than multiple sclerosis, and spinal injury caused by myelitis could be a reasonable explanation.^[1]^ Urgency, nocturia, urinary incontinence, and voiding symptoms were reported as most frequent symptoms.^[1]^ Urinary tract infection was diagnosed in our case during hospitalization and follow-up, and it could be related to voiding symptoms.

Hyponatraemia could be caused from multiple factors, and one of infamous causes being the SIADH, which could possibly be caused from disorders of the central nervous system.^[13]^ However, only rare cases mentioned the association of the SIADH with NMOSD.^[2]^ Besides, other autoimmune comorbidities such as Sjögren syndrome, systemic lupus erythematous, and mixed connective tissue disease could present due to circulating autoantibodies.^[14]^ It was speculated that bladder distension induced by chronic urinary retention could cause stimulation of ADH release from the posterior pituitary.^[15]^ Our case only experienced AUR for 2 days; therefore, SIADH could be more likely related to demyelination of central nervous system.^[2]^

Elevation in prolactin level in NMO patient has also been reported, and the level would significantly decrease during remission period.^[16]^ Elevation in prolactin level was also found in our case in first time diagnosed and during relapsing, and prolactin data could be followed up again later in the future.

To the best of our knowledge, this was the first childhood case of NMO accompanied by refractory hyponatremia. Overall, our findings suggest that in childhood cases presenting with refractory hyponatremia and limb weakness, NMO or NMOSD should be considered possible diagnoses despite their rarity in pediatric cases.

## Acknowledgments

This work was supported by the Ministry of Science and Technology, Taiwan, Republic of China (MOST106-2314-B-016-041-MY3 and MOST109-2314-B-016-023 to S.-J.C.) and by a research grant from Tri-Service General Hospital, Taiwan, Republic of China (TSGH-C107-008-S03 to S.-J.C.). In addition, a part of research grants from Cheng Hsin General Hospital, Taiwan, Republic of China (CH-NDMC-107-05 to S.-J.C.).

## Author contributions

**Conceptualization:** Po-Chang Hsu, Po-Wei Wu, Sheng-Yuan Ho.

**Data curation:** Jhao-Jhuang Ding.

**Investigation:** Chiung-Hsi Tien, Chih-Fen Hu, Chia-Hsiang Yu.

**Supervision:** Shyi-Jou Chen.

**Validation:** Hung-Hsiang Fang.

**Writing – original draft:** Tai-Han Lin.

**Writing – review & editing:** Chia-Cheng Sung.
